# Complete Genome Assembly of Yersinia pestis subsp. *pestis* bv. Medievalis SCPM-O-B-6530, a Proline-Dependent Strain Isolated from the Central-Caucasian High-Mountain Plague Focus in Kabardino-Balkar Republic (Russia)

**DOI:** 10.1128/mra.01115-21

**Published:** 2022-01-06

**Authors:** Angelina A. Kislichkina, Elizaveta M. Mazurina, Mikhail E. Platonov, Yury P. Skryabin, Angelika A. Sizova, Viktor I. Solomentsev, Elena V. Galkina, Alexandra S. Trunyakova, Tat’yana V. Gapel’chenkova, Svetlana V. Dentovskaya, Alexandr G. Bogun, Andrey P. Anisimov

**Affiliations:** a State Research Center for Applied Microbiology and Biotechnology, Obolensk, Russia; b Pushchino State Institute of Natural Science, Pushchino, Russia; University of Maryland School of Medicine

## Abstract

We report the complete genome assembly of Yersinia pestis subsp. *pestis* bv. Medievalis SCPM-O-B-6530, a strain belonging to the most ancient phylogenetic group (group 2.MED0) of Y. pestis subsp. *pestis* bv. Medievalis. This proline-dependent strain, carrying an additional plasmid (pCKF), was isolated from the Central-Caucasian high-mountain plague focus in Kabardino-Balkar Republic, Russia.

## ANNOUNCEMENT

Representatives of two phylogroups of Yersinia pestis biovar Medievalis strains circulate in the Central-Caucasian high-mountain plague focus in Russia. The most ancient of them, strains belonging to phylogenetic group 2.MED0, are characterized by reduced virulence, unusual proline dependence, and the presence of an additional 5.4-kb plasmid (pCKF) (1–4). Comparison of such partially attenuated endemic strains with classic highly virulent pandemic strains can help to identify new promising molecular targets for the prevention and treatment of plague. Here, we report the complete genome sequence of Yersinia pestis subsp. *pestis* bv. Medievalis strain SCPM-O-B-6530, which was isolated from Citellophilus tesquorum fleas collected at the entrances to gopher (Spermophilus musicus) burrows in the Central-Caucasian high-mountain natural plague focus in Kabardino-Balkar Republic (Russia) in 2000. Chopped fleas were suspended in phosphate-buffered saline and plated on Hottinger agar supplemented with lysed lamb’s blood (1%) and gentian violet (1:100,000) for isolation of Yersinia pestis. The primary identification was confirmed by microscopic examinations and biochemical identification tests.

The stocks of strains were stored at –70°C in cryoprotective medium. The bacteria were cultured on brain heart infusion agar (HiMedia, India) for 18 h at 28°C, and DNA samples were extracted using a DNA minikit (BioFact, Daejeon, Republic of Korea) for both sequencing technologies. Genome sequencing was completed using Oxford Nanopore Technologies and Illumina platforms. For the Illumina MiSeq instrument, DNA libraries were prepared using the Nextera DNA library preparation kit and the MiSeq reagent kit v3 (300 cycles). Whole-genome sequencing was performed following the manufacturer’s protocols. A rapid barcoding kit (RBK004) and MinION flow cell (R9.4.1) were used for Oxford Nanopore Technologies MinION sequencing. Whole-genome sequencing was performed by running MinKNOW software v18.05.5 (time, 48 h; 180 mV), and base calling was performed using Albacore v2.3.1 (Oxford Nanopore Technologies) with default settings.

Up to 504,785 paired-end reads (125,284,863 bases) were generated by Illumina sequencing, and 95,035 long single-end reads, with an average length of 4,461 bp (total count, 424,022,177 bp), were generated by MinION sequencing, with a total coverage depth of 258-fold. The hybrid genome was assembled from all reads using Unicycler v0.4.7 with default settings, which included primary filtering and quality control (5). The resulting assembly included five circular contigs. The contigs were classified as a chromosome or plasmids using BLAST v2.9.0 with the nonredundant database (nucleotide search) with default settings (6).

The final genome assembly contained one chromosome and four plasmids; the molecules were determined to be complete and were rotated to a certain base by Unicycler v0.4.7 with default settings. The chromosome was rotated to *dnaA*, and the plasmids were rotated to the replication genes. The genome contained 4.78 Mb, with a G+C content of 47.69%. In detail, the chromosome has 4,596,548 bp, plasmid pCD has 70,503 bp, plasmid pMT has 101,698 bp, plasmid pPCP has 9,611 bp, and plasmid pCKF has 5,400 bp. The chromosome and the plasmids were annotated with the NCBI Prokaryotic Genome Annotation Pipeline (PGAP) v4.9 (7). This strain has a total of 4,312 genes, including 4,015 protein-coding sequences, 22 rRNA genes, 72 tRNA genes, and 192 pseudogenes.

This study will facilitate the understanding of the genomic variability of Y. pestis and will provide an opportunity for comparison with other strains.

### Data availability.

The genome sequences for Y. pestis C-771 have been deposited in GenBank under accession no. CP045158.1 for the chromosome and accession no. CP045159.1, CP045160.1, CP045161.1, and CP045162.1 for the plasmids. The raw sequence reads have been deposited in the SRA under accession no. SRR5641454 (MiSeq reads) and SRR10259782 (MinION reads).
